# Field-free manipulation of magnetization alignments in a Fe/GaAs/GaMnAs multilayer by spin-orbit-induced magnetic fields

**DOI:** 10.1038/s41598-017-10621-6

**Published:** 2017-08-31

**Authors:** Sangyeop Lee, Taehee Yoo, Seul-Ki Bac, Seonghoon Choi, Hakjoon Lee, Sanghoon Lee, Xinyu Liu, Margaret Dobrowolska, Jacek K. Furdyna

**Affiliations:** 1 Physics Department, Korea University, Seoul, 136-701 Republic of Korea; 2 Physics Department, University of Notre Dame, Notre Dame, IN 46556 USA

## Abstract

We investigate the process of selectively manipulating the magnetization alignment in magnetic layers in the Fe/GaAs/GaMnAs structure by current-induced spin-orbit (SO) magnetic field. The presence of such fields manifests itself through the hysteretic behavior of planar Hall resistance observed for two opposite currents as the magnetization in the structure switches directions. In the case of the Fe/GaAs/GaMnAs multilayer, hystereses are clearly observed when the magnetization switches direction in the GaMnAs layer, but are negligible when magnetization transitions occur in Fe. This difference in the effect of the SO-field in the two magnetic layers provides an opportunity to control the magnetization in one layer (in the presence case in GaMnAs) by a current, while the magnetization in the other layer (i.e., Fe) remains fixed. Owing to our ability to selectively control the magnetization in the GaMnAs layer, we are able to manipulate the relative spin configurations in our structure between collinear and non-collinear alignments simply by switching the current direction even in the absence of an external magnetic field.

## Introduction

Spintronic devices often involve magnetic multilayers in which either giant magnetoresistance (GMR) or tunneling magnetoresistance (TMR) are used for information storage and processing^1–4^. These effects typically arise from different magnetization alignments between magnetic layers in the multilayer. The ability to independently control the magnetization of one layer relative to the other is therefore of key importance for operating such multilayer-based spintronic devices. The most common approach to achieve this is by designing structures with magnetic layers that have significantly different magnetic coercivities, so that the magnetization of the soft magnetic layer can be manipulated, while that of the hard magnetic layer remains fixed. The hard magnetic layer in such structures is commonly achieved by forming a ferromagnet/antiferromagnet (FM/AFM) bilayer, in which the coupling between the two layers pins the magnetization in the FM layer along a fixed direction^5^. This technique has been used, for example, in recently commercialized spin transfer torque (STT) magnetic random access memory (MRAM) devices^6–9^, in which the magnetization of the soft magnetic layer is controlled by the STT^10–12^ generated by a spin-polarized current. However, involving additional antiferromagnetic layers in such devices requires complex fabrication steps and makes device structures more complicated. It is therefore desirable to have simpler and more elegant multilayer systems, in which the magnetization alignment in one layer can be selectively manipulated with respect to other layers by an electric current.

Recently, the ability to generate current-induced magnetic fields originating from spin-orbit interaction (SOI) was demonstrated in crystalline magnetic films such as GaMnAs^13, 14^, in which inversion symmetry is broken either by the crystal structure itself or by strain^15–17^. This spin-orbit (SO)-field has been shown to be sufficiently strong for rotating the magnetization in certain materials^18–20^. Additionally, unlike STT devices^10–12^, the SO-field does not require spin-polarized currents to achieve reorientation of magnetization. Such SO-field thus provides a powerful and convenient new method for electrically controlling magnetization in ferromagnetic films. By choosing an appropriate combination of magnetic films, this effect can then be used to manipulate the magnetization of a specific layer in a multilayer while leaving the magnetization of other magnetic layer unchanged.

The aim of this paper is to explore such manipulation of magnetization alignments by SO fields experimentally. For this purpose, we have designed a hybrid structure consisting of a GaMnAs ferromagnetic semiconductor and a Fe ferromagnetic metallic layer. Since these two layers experience SO-fields differently at a given current, we can then selectively control the magnetization of one magnetic layer relative to the other magnetic layer in this structure. We will further show that such selective manipulation of magnetization of the GaMnAs layer allows us to control the magnetization alignments in the multilayer between collinear and non-collinear configurations even in the absence of an external magnetic field.

## Methods

### Sample growth

**Figure 1:**
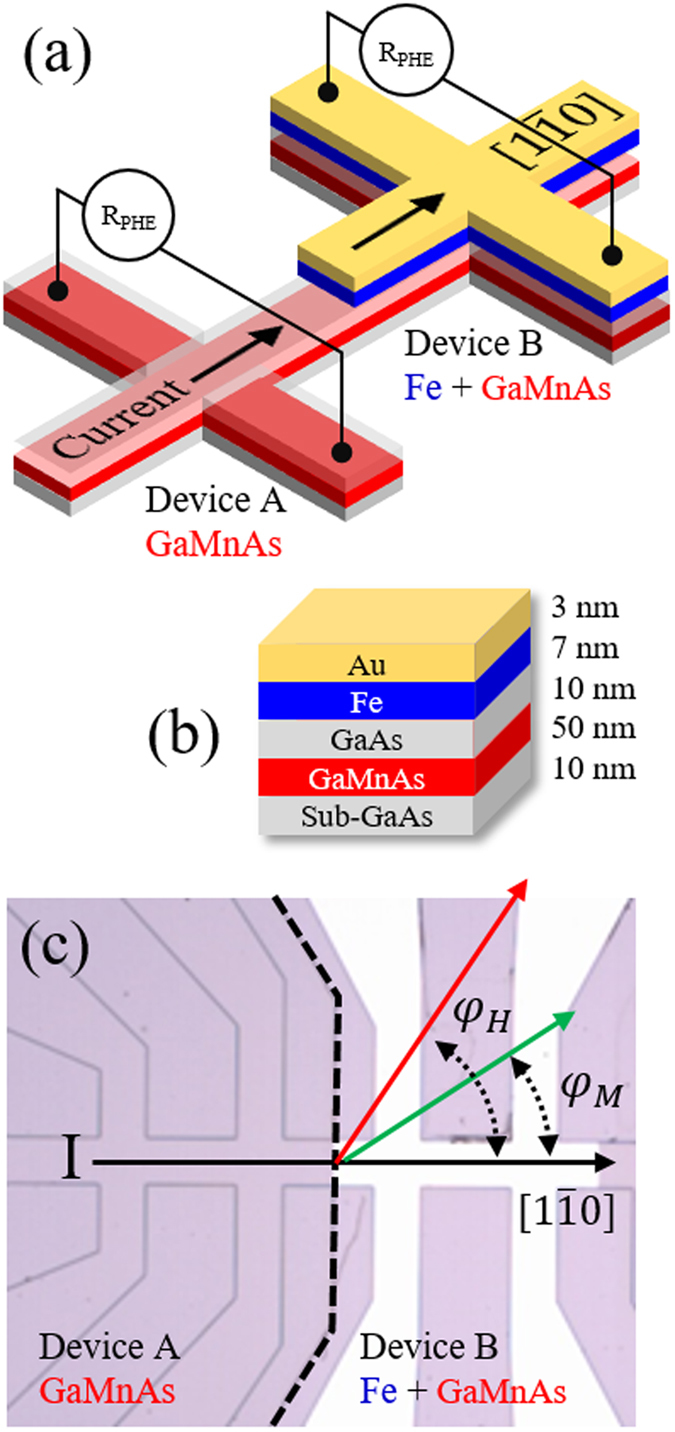
(**a**) Schematic diagram of the Hall device. (**b**) Structure of Fe/GaAs/GaMnAs tri-layer grown on a GaAs substrate. (**c**) Top view of Hall devices used for PHR measurements patterned on the GaMnAs region (Device A) and on the Fe/GaAs/GaMnAs multilayer region (Device B). Directions of positive current, external magnetic field, and magnetization are shown by arrows.

The Fe/GaAs/GaMnAs ferromagnetic hybrid structure designed for this investigation was grown by molecular beam epitaxy (MBE) on a (001) GaAs substrate. Prior to growth of the GaMnAs layer, a 100 nm GaAs buffer layer was deposited on the substrate at 600 °C. A 50 nm Ga_1−*x*_Mn_*x*_As film with *x* = 0.06 was then grown on top of the GaAs buffer, followed by deposition of a 10 nm GaAs spacer layer at 250 °C. The system was then cooled to room temperature for epitaxial deposition of a crystalline Fe layer. Finally, the structure was and capped by a 3 nm Au film to protect the Fe layer from oxidation. The complete structure is shown schematically in Fig. 1(b).

### Device fabrication

In order to investigate the behavior of the two magnetic layers independently, the sample was divided into two regions of size 5 × 5 mm^2^, and one of the regions was then selectively etched to remove the top Fe layer. This provides the opportunity to simultaneously investigate the behavior of the single GaMnAs layer and the Fe/GaAs/GaMnAs trilayer at identical experimental conditions, and thus to independently identify the effect of the SO field in GaMaAs and in Fe. For transport measurements, a $1000\times 50$ μm^2^ Hall device was patterned on each of these two regions by photolithography and dry etching, with the long dimension along the $[1\bar{1}0]$ direction. A schematic view and an optical image of the device are shown in Fig. 1(a) and (c), respectively. In what follows, the Hall devices patterned from the single GaMnAs layer and from the Fe/GaAs/GaMnAs trilayer will be referred to as device A and B, respectively.

### Transport experiments

Planar Hall resistance measurements were performed using a sample holder which allows the magnetic field to be applied at arbitrary directions in the plane of the sample. The electromagnet used for this purpose was mounted on a rotating table, so that the field could either be swept along an arbitrary fixed direction, or could be continuously rotated in the film plane at a fixed field magnitude. In this study we will refer to the current flowing in the $[1\bar{1}0]$ direction as positive, and in the $[\bar{1}10]$ direction as negative. The directions of the applied magnetic field *φ*_*H*_ and of the magnetizations of the magnetic layers *φ*_*M*_ are measured counterclockwise from the $[1\bar{1}0]$ crystallographic direction in the sample plane (i.e., from the positive current direction of the Hall device, as shown in Fig. 1(c)).

**Figure 2:**
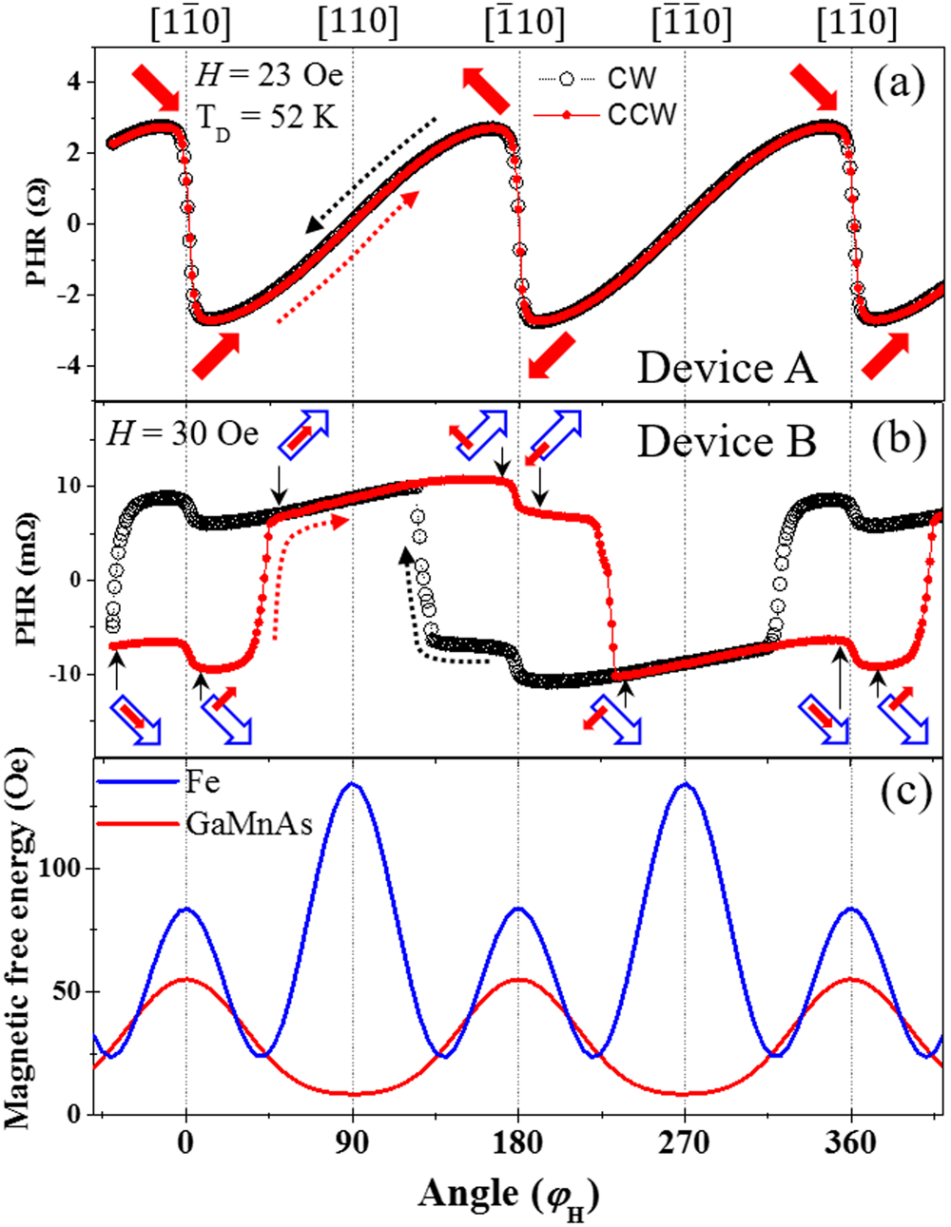
PHR data measured with field strengths of 23 Oe and 30 Oe for device A (panel (a)) and device B (panel (b)). The red (solid) and black (open) symbols represent data taken with CCW and CW rotation of the field, respectively. Directions of magnetization are shown with red (solid) and blue (open) arrows at corresponding angular positions. Magnetic free energy density obtained for GaMnAs (red curves) and Fe layers (blue curves) for a current of 3.36 mA are shown in panel (c).

The temperature of the sample holder was set at 3 K during the measurements. However, it is known that Joule heating by the current flowing in the GaMnAs device changes its temperature during the measurement^21, 22^. The current of 3.36 mA used in this study, which corresponds to current densities of ~1.34 × 10^5^ A/cm^2^, is indeed sufficiently large to generate significant Joule heating and to increase the temperature of the Hall device to ~52 K [see Supplementary Material 3]. The magnetic anisotropies of the GaMnAs and the Fe layers, which govern the process of magnetization reversal, are then expected to change due to this increase of temperature^23–25^. We have therefore investigated the magnetic anisotropy of the GaMnAs and the Fe layers^26–28^ using several different values of the current [Supplementary Material 1]. The magnetic free energy profiles obtained for the GaMnAs and the Fe layers at 3.36 mA are shown in Fig. 2(c). The switching of magnetization in the GaMnAs and the Fe layers is governed by those magnetic energy density profiles in our experiments.

## Results

To demonstrate the process of selective control of magnetization we use currents of opposite polarity, and we monitor the resulting behavior of the device by planar Hall effect (PHE) using experimental arrangement shown in Fig. 1(a). The device in the left (designated as device A) and in the right side (designated as device B) are fabricated from GaMnAs and Fe/GaAs/GaMnAs layers, respectively, obtained by selectively etching the sample shown in Fig. 1(b) (fabrication details are described in Methods). It is well known that planar Hall resistance (PHR) is given by ref. 29

1 $${R}_{{\rm{P}}{\rm{H}}{\rm{R}}}=\frac{k}{t}{M}^{2}\,\sin \,2{\phi }_{M},$$

where *k* is a constant related to the anisotropic magnetoresistance; *M* is the magnetization of the film; *t* is the film thickness; and φ_*M*_ is the direction of the magnetization as defined in Fig. 1(c). Owing to the sensitive dependence of PHR on the direction of magnetization, the angular dependence of PHR measured by rotating the external field direction φ_*H*_ in the sample plane at a constant field strength is one of the best techniques for investigating the behavior of magnetization, including its reorientation. As will be seen, this high sensitivity will be especially useful in detecting the effects of SO fields in our magnetic multilayer^30, 31^. Furthermore, the anisotropic magnetoresistance constants *k* in GaMnAs and in Fe layers are known to have opposite signs, which will enable us to distinguish the rotation of magnetization in the two magnetic layers in our experiment^32–34^.

Figure 2(a) and (b) show PHR data for devices A and B obtained with magnetic fields of 23 Oe and 30 Oe, respectively, using a current of 3.36 mA. For this measurement, the magnetization of the film was initially set at the energy minimum in the 4^th^ quadrant by a field of 2000 Oe applied along φ_*H*_ = −40°. The field was then reduced to 23 Oe (or 30 Oe), and PHR was measured as the field was rotated at this fixed value between φ_*H*_ = −40° and φ_*H*_ = 400°. In Fig. 2 the open symbols are used for PHR results obtained with clockwise (CW) rotation, and solid symbols are for counterclockwise (CCW) rotation.

PHR data from device A (the single GaMnAs layer) plotted in Fig. 2(a) show abrupt transitions at φ_*H*_ = 0°, 180° and 360°, while showing a gradual variation as the field rotates across φ_*H*_ = 90° and 270°. This is a typical angular dependence of PHR for GaMnAs films with a strong uniaxial anisotropy contribution along the [110] and $[\bar{1}\bar{1}0]$ directions^23, 25, 35^ Thus, even though cubic anisotropy creates four energy barriers at all four < 110 > orientations in the film, the presence of the uniaxial anisotropy significantly lowers the barriers at [110] (φ_*H*_ = 90$^\circ$) and $[\bar{1}\bar{1}0]$ (φ_*H*_ = 270°). This is seen in the magnetic anisotropy energies calculated for device A (see Supplementary Material 1), plotted in red in Fig. 2(c), where the energy barriers at [110] and $[\bar{1}\bar{1}0]$ are shown to be almost negligible. Directions of magnetization corresponding to PHR values observed during the rotation of the field are indicated by red thick arrows in Fig. 2(a).

The PHR data obtained for device B (i.e., Fe/GaAs/GaMnAs multilayer) are plotted in Fig. 2(b), and show a much more complex behavior owing to the effects of magnetization realignments in the GaMnAs and Fe layers as the field is rotated in the sample plane. In addition to transitions of magnetization at φ_*H*_ = 0°, 180° and 360° originating from reorientations of magnetization in the GaMnAs layer, there are now distinct sharp transitions that result in additional hystereses at φ_*H*_ = 0°, 180°, and 360°. These transitions, showing distinct broad hystereses, arise from the Fe layer, which has two orthogonal in-plane magnetic easy axes due to its cubic anisotropy, as shown in blue in Fig. 2(c). Magnetization orientations occurring during the CCW rotation of the field are shown as solid red arrows in Fig. 2(b) for the GaMnAs layer, and as open blue arrows for the Fe layer. Importantly, this shows that the magnetization alignments in the two magnetic films change from collinear to non-collinear during the rotation of the field.

Note that changes in PHR corresponding to magnetization transitions in the Fe layer occur only between the 4^th^ and the 1^st^ quadrants, as shown by open blue arrows during the 360° field rotation. This is due to the small value of the magnetic field (30 Oe), which is not sufficiently high to overcome the strong magnetic anisotropy barrier of the Fe layer at the [110] direction, as shown by the blue curve in Fig. 2(c). The magnetization of the Fe layer therefore experiences only 90° change during the rotation of field direction over 360°. Note that a full rotation of magnetization in the Fe layer can be observed when a larger external field is used, as shown in Supplementary Material 2.

Another notable fact in the data shown in Fig. 2 is that the magnitude of PHR in GaMnAs (i.e., in device A) is quite different from that in the Fe/GaAs/GaMnAs structure (i.e., in device B). This difference arises from the different values of film thickness, anisotropy constants, and magnetization, all of which are parameters in Eq. (1). These parameters are clearly different for the two devices, since device A is fabricated from a single GaMnAs layer, while device B consists of a Fe/GaAs/GaMnAs trilayer structure. One may also wonder whether the effect of interlayer exchange coupling plays a role in the magnetization switching in the Fe/GaAs/GaMnAs system. We have checked for the presence of such coupling in our system by performing minor loop scan experiments^36^, in which the magnetization of only the GaMnAs layer is switched, while that of the Fe layer remains fixed. By observing the absence of a shift in the minor hysteresis loop (see Supplementary Material 6), we have confirmed that the interlayer exchange coupling between GaMnAs and Fe layer is negligibly small in our system.

**Figure 3:**
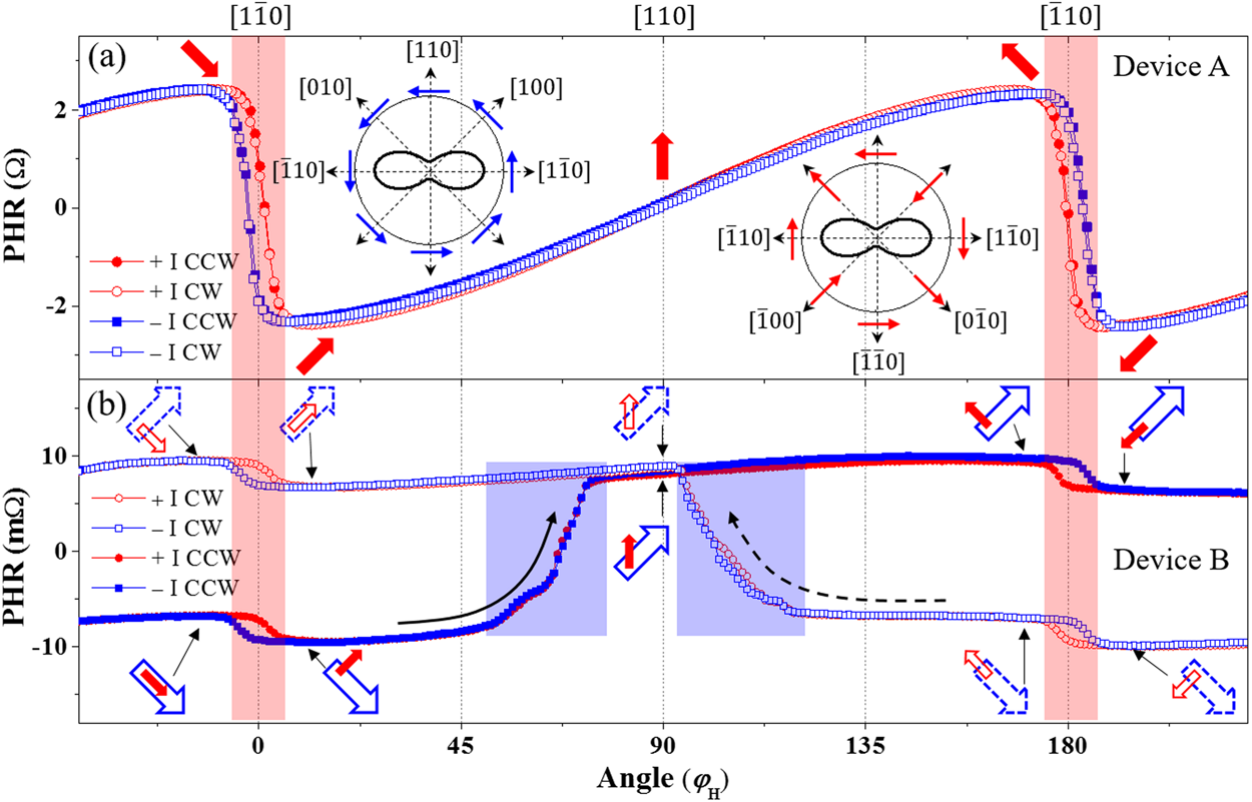
Angular dependence of PHR measured with two opposite current directions for devices A (panel a) and B (panel b) as a 23 Oe field is rotated. Open symbols are for data obtained with CW rotation; solid symbols for CCW rotation; circles correspond to positive current; squares to negative current. Directions of magnetization in GaMnAs and Fe layers are shown with red solid and blue open arrows at corresponding positions. The data show clear hystereses between positive and negative currents at φ_*H*_ = 0°and 180°, where magnetization of the GaMnAs layer makes an abrupt transition (see regions shaded in red). Directions of the SO-fields originating from broken inversion symmetry of the GaMnAs crystal structure and from strain are shown in the left and right insets in the top panel. Solid arrows (red and blue) in the insets represent the SO fields, and dotted (black) arrows show the current directions.

The presence of current-induced SO-field – the key object of this paper – is identified by measuring the angular dependence of PHR with driving currents of opposite polarities. For these measurements, we follow the same initializing process as already described, and we measure the PHR with several different current densities as a 23 Oe field is rotated CCW over 360°. The same measurement is then repeated with opposite current polarity. The strength of SO-field obtained from the hysteresis widths are in the order of 1.0 Oe for the current density of ~10^5^ A/cm^2^. This range of the SO-field is consistent with that obtained by 2^nd^ harmonic Hall measurements (see Supplementary Material 4)^37^. The two sets of PHR data obtained with currents of ±3.36 mA are plotted with solid symbols (circles for positive and squares for negative currents) in Fig. 3. These current-polarity-dependent measurements are then repeated for CW field rotation, and the results for the latter case are plotted as open symbols (circles for positive and squares for negative currents) in Fig. 3.

The PHR data for devices A and B are plotted in Fig. 3(a) and (b), respectively. The results for device A (single GaMnAs epilayer) look nearly the same as those in Fig. 2(a) for each current direction. However, they clearly show hystereses between PHR data obtained with positive and negative currents across φ_*H*_ = 0° and 180°, i.e., across $[1\bar{1}0]$ and $[\bar{1}10]$ field orientations shown by regions shaded in red. This emergence of hystereses between data obtained with two opposite current directions signals the presence of current-induced SO-fields. It is well established that broken inversion symmetry in the crystal structure (i.e., the Rashba-type SO-field) and distortion of crystalline bonds due to strain (i.e., strain-induced SO-field) are two major mechanisms underlying the SO-fields in GaMnAs^15–17^. The left and right insets in Fig. 3(a) show the directions of the Rashba-type and the strain-induced SO-fields, respectively. The fact that the transition occurs at a smaller angle for the negative current than for the positive current in the hysteresis around $[1\bar{1}0]$ (this behavior is reversed in the hysteresis at the $[\bar{1}10]$ direction) indicates that the strain-induced SO-field in the GaMnAs layer is larger than its Rashba-type counterpart^17^. The result observed in device A thus demonstrates that current-induced SO fields indeed exist in the GaMnAs layer, and can thus be used to manipulate magnetization of GaMnAs by current polarity.

However, in our experiment we are not able to distinguish between the contributions of Rashba-type and strain-induced-type SO-fields. In order to separate the contribution of these two types of fields, one needs to carry out experiments as a function of current direction, which can show either additive, non-additive, or subtractive contributions of the fields, as seen in the insets in Fig. 3. Such experiments have indeed been performed on GaMnAs films by other groups^15, 17^, and have shown that the Rashba-type SO-field and the strain-induced SO-field contribute in approximately a 1:3 ratio in this material^17^. In our case, however, the Hall device is fabricated along the $[1\bar{1}0]$ direction, allowing us to investigate the SO field with current applied in only one direction. Since the main focus of our study is on current-induced manipulation of relative magnetic alignment in our Fe/GaAs/GaMnAs structure, we must therefore restrict ourselves to a study of magnetization switching induced by the specific combination of SO fields associated with that direction of current flow.

The current-polarity-dependent PHR observed for device B is shown in Fig. 3(b). The PHR values obtained with CCW (solid symbols) and CW field rotations (open symbols) are now well separated in the vertical direction. This is due to the magnetization of the Fe layer, which makes different contributions to PHR as its orientation changes: as shown in Eq. (1), when φ_*M*_ is in the 1^st^ quadrant, the magnetization in the Fe layer makes a positive contribution to PHR; and the contribution is negative for φ_*M*_ in the 4^th^ quadrant. Nevertheless, rotations of the magnetizations are clearly observed in both magnetic layers, as indicated by solid arrows for CCW and by dotted arrows for CW rotations. Note, however, that a hysteresis between the positive and the negative currents only appears at magnetization transitions in the GaMnAs layer (see red shaded regions), and is absent for magnetization transitions in the Fe layer (see blue shaded region). This indicates that the effect of SO-fields in the Fe layer is negligible compared to that in the GaMnAs layer. This large difference in the SO fields between the two magnetic layers automatically provides an opportunity for selectively manipulating the orientation of magnetization by an applied current in one of the layers comprising the structure (in the present case, GaMnAs), while the magnetization of the other layer (in the present case, Fe) remains fixed.

**Figure 4:**
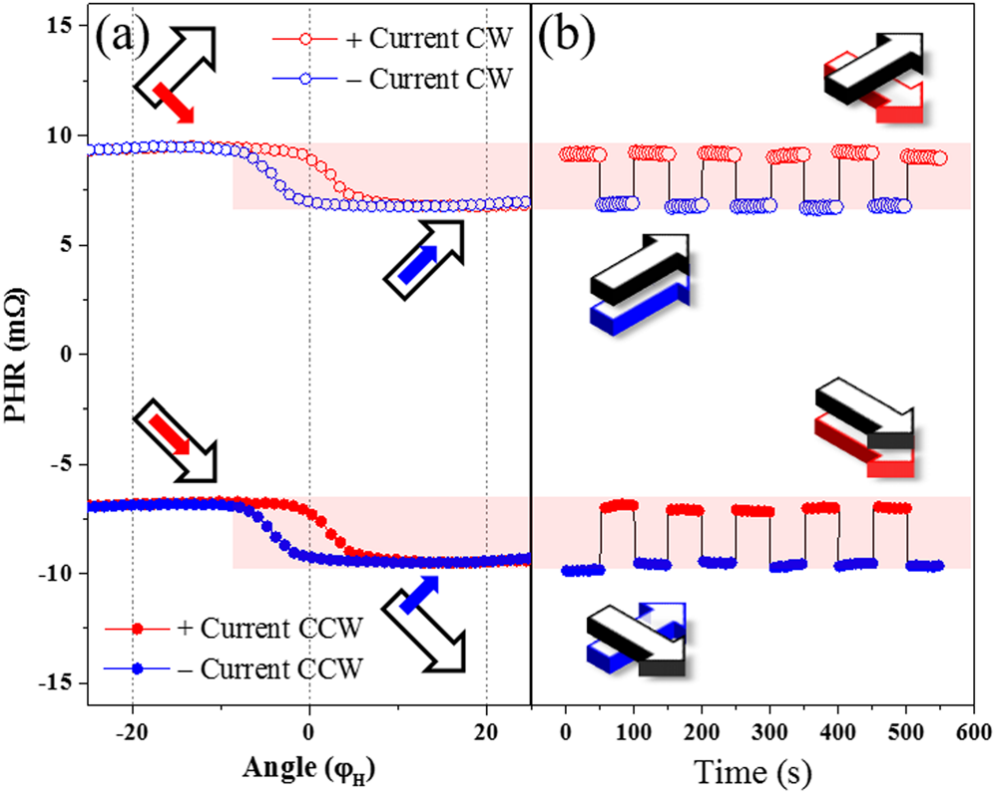
Left panels: expanded view of angular dependence of PHR shown in Fig. 3(b) for φ_*H*_ between −25° to +25°. Right panels: PHR measured as a function of time, showing abrupt switching of PHR as current polarity is reversed every 50 seconds. Magnetization alignments for the two PHR states corresponding to top and bottom of the hysteresis loops in left panels are schematically shown in the insets. In both panels the magnetization for the Fe layer is shown by black arrows, and for GaMnAs in color, red for positive and blue for negative current.

In order to demonstrate such selective manipulation of magnetization in our structure, we now focus on the hysteresis appearing near φ_*H*_ = 0° (i.e., when the applied field is near the $[1\bar{1}0]$ direction). The left panels of Fig. 4 are replots of the data shown in Fig. 3(b) for φ_*H*_ between −25° and +25°. Let us first consider the case for CCW rotation shown in solid symbols in Fig. 4(a). In this case we set the field of 23 Oe at the $[1\bar{1}0]$ direction (i.e., ${\phi }_{H}=0^\circ$), where the hysteresis occurs between positive and negative current directions, and we measure the PHR with a negative current of -3.36 mA for 50 seconds. The resulting data are plotted as blue solid circles in Fig. 4(b), showing a constant value of −9.5 mΩ. This is the PHR value measured with a negative current at the ${\phi }_{H}=0^\circ$ position, corresponding to the low hysteresis level in Fig. 4(a). As schematically shown in the lower left inset of Fig. 4(b), at this point the magnetization alignment between GaMnAs and Fe layers is nearly perpendicular. The polarity of the current was then switched from negative to positive. As soon as the polarity of the current is reversed, the PHR shows an abrupt jump to -7.0 mΩ (i.e., to the high level of hysteresis) in Fig. 4(a). This change of PHR indicates a rotation of magnetization in the GaMnAs layer from the 1^st^ quadrant to the 4^th^ quadrant, resulting in a nearly parallel magnetization alignment in the GaMnAs and the Fe layers, as schematically shown in the lower right inset of Fig. 4(b).

When the polarity of the current is switched back to positive, the PHR again makes an abrupt transition to the low value that was observed at the initial state. We have reversed the direction of the current repeatedly to test the reproducibility of such transitions, and PHR was observed to switch systematically between the high and the low values as the current changed sign, as shown in Fig. 4(b), maintaining a constant value until the current polarity was changed again. This indicates that the SO field produced by the current switches the magnetization of the GaMnAs layer between the stable magnetic energy minima in the 1^st^ and in the 4^th^ quadrants, while the magnetization in the Fe layer remains unchanged in the 4^th^ quadrant. The same current-dependent switching can be done after CW rotation of the applied field, as shown with open symbols in Fig. 4. In this case, however, the magnetization of the GaMnAs layer switches between 4^th^ and 1^st^ quadrants, while that of the Fe layer remains fixed in the 1^st^ quadrant. The magnetization alignments in the two layers of the structure corresponding to the two observed values of PHR are shown in the insets of Fig. 4(b).

These experiments clearly show that the reversal of the SO field produced by reversing the direction of the current switches the magnetic alignments from nearly parallel to nearly perpendicular configurations, as shown by arrows in the insets of Fig. 4(b). Additionally, we performed magnetization switching experiments as a function of the applied current in a constant background field of *H* = 20 Oe along the $[1\bar{1}0]$ direction, which shows switching of PHR near ±2.9 mA (see Supplementary Material 5). These experiments show that during the process of magnetization transition the PHR shows intermediate values, which indicate formation of multi-domains structures. From this we infer that the reorientation of magnetization in GaMnAs layer occurs via nucleation of domains and their propagation^36, 38^.

**Figure 5:**
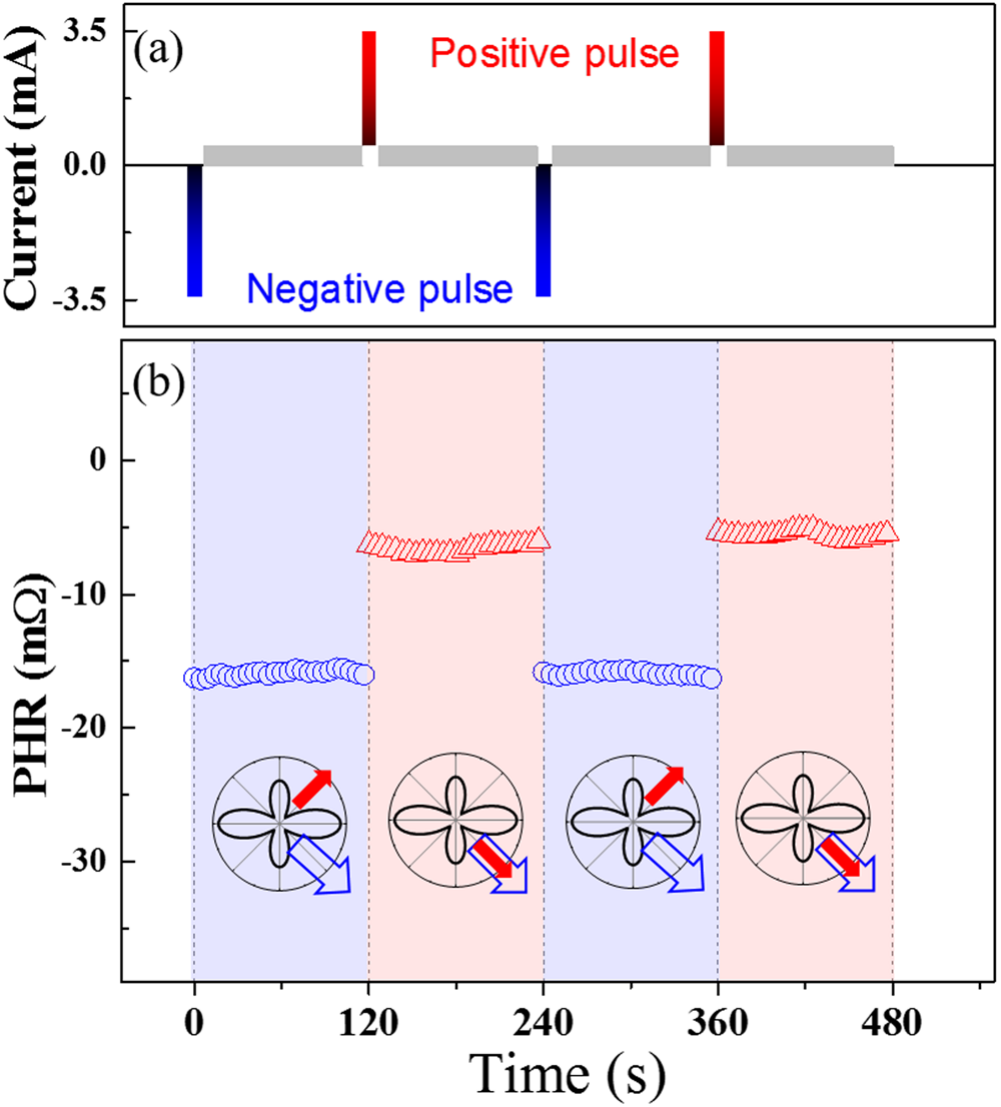
PHR measured after application of current pulses as a function of time, showing a switching of PHR as the polarity of the current pulse is reversed. Magnetization alignments for the two PHR states corresponding to high and low PHR values are schematically shown by the insets. Directions of magnetization are shown with red (solid) arrows for GaMnAs, and with blue (open) arrows for the Fe layer.

Finally, we show that magnetization switching in the GaMnAs layer can be achieved by using only a current pulse, without assistance of an external field. For this experiment we use current pulses with a magnitude of 3.48 mA, which is sufficiently large to eliminate complications from the formation of multi-domain structures during magnetization reorientation. We then use a continuous current of 0.5 mA for detecting the direction of magnetization by PHR measurements. The switching behavior obtained in this manner is shown in Fig. 5, where the top panel shows the current pulses, and the bottom panel shows the corresponding PHR values. Specifically, after initializing the magnetization of both layers in the 4^th^ quadrant, a negative current pulse of 3.48 mA was applied for 3 s in the absence of an external field. PHR was then measured with a weak sensing current of 0.5 mA for 120 s, as shown by blue circles in the lower panel of Fig. 5. A negative current pulse, which generates an SO field along the [110] direction, causes the magnetization of the GaMnAs layer to re-orient to the energy minimum in the 1^st^ quadrant, while the magnetization of Fe remains in the 4^th^ quadrant. The magnetic alignment for this state is shown below the corresponding PHR data as an inset, where the red solid and blue open arrows indicate directions of magnetization in the GaMnAs and the Fe layers, respectively.

A positive current pulse was then applied for 3 s, and the PHR values that followed are shown as red triangles in Fig. 5. As a result of the current pulse, the magnetization in the GaMnAs layer rotates to the energy minimum in the 4^th^ quadrant, as shown by the corresponding inset, causing the PHR value to abruptly jump to a smaller magnitude (i.e., toward zero). However, the value of PHR remains negative due to the magnetization of the Fe layer in the 4^th^ quadrant, which gives a negative PHR value. The switching behavior between the two PHR values was systematically observed as subsequent current pulses of opposite polarity were applied, as seen in Fig. 5, showing that the field-free switching phenomenon is reproducible. These results clearly demonstrate that magnetic alignments in our structure can be controlled by current pulses alone, thus providing a significant advantage for operating a spintronic device.

In summary, we have demonstrated that electric current can be used to control magnetization alignment in the ferromagnetic layers of a Fe/GaAs/GaMnAs multilayer. Angle-dependent PHR measurements on this structure revealed that diverse magnetization alignments in the two magnetic layers, including collinear and non-collinear configurations, can be obtained by changing the polarity of the current through the multilayer. The mechanism underlying this process is the presence of a current-induced SO field, which reverses as the current is reversed. The presence of the SO field was identified in our angle-dependent PHR measurements by the presence of a clear hysteresis in PHR observed with two opposite current directions. However, the hystereses were only observed near the $[1\bar{1}0]$ and $[\bar{1}10]$ field orientations, where abrupt transition of magnetization occur in the GaMnAs layer, indicating that the SO field acts only in the GaMnAs layer, and is negligible in the Fe layer. This indicates that magnetization can be manipulated by the SO-field in the GaMnAs layer, while it remains fixed in the Fe layer. Such selective rotation of magnetization in the GaMnAs layer relative to that of the Fe layer in turn results in different values of PHR. Furthermore, our experiments show that such switching of magnetic alignments in Fe/GaAs/GaMnAs multilayers can be achieved by current pulses alone, without the presence of applied magnetic fields, and can thus be employed in current-controlled spintronic devices.

## Electronic supplementary material

Field-free manipulation of magnetization alignments in a Fe/GaAs/GaMnAs multilayer by spin-orbit-induced magnetic fields
