# Supplementary material for: Field-free manipulation of magnetization alignments in a Fe/GaAs/GaMnAs multilayer by spin-orbit-induced magnetic fields
Source: Sci Rep. 2017 Aug 31;7:10162. doi: 10.1038/s41598-017-10621-6 (PMC5578966; doi:10.1038/s41598-017-10621-6)
Supplement: Supplementary file 1 — Field-free manipulation of magnetization alignments in a Fe/GaAs/GaMnAs multilayer by spin-orbit-induced magnetic fields [file 41598_2017_10621_MOESM1_ESM.pdf]

Field-free manipulation of magnetization alignments in a Fe/GaAs/GaMnAs multilayer by spin-orbit-induced magnetic fields

- Supplementary Material -

Sangyeop Lee[1], Taehee Yoo[1], Seul-Ki Bac[1], Seonghoon Choi[1], Hakjoon Lee[1], Sanghoon Lee*[1], Xinyu Liu[2], M. Dobrowolska[2], and Jacek K. Furdyna[2]

[1]*Physics Department, Korea University, Seoul 136-701, Republic of Korea*

[2]*Physics Department, University of Notre Dame, Notre Dame, IN 46556, USA*

Supplementary 1

It is well established that magnetic anisotropy of a GaMnAs film varies with temperature.[1] This leads to the dependence of the magnetic anisotropy on the current flowing through the film due to Joule heating, which changes sample temperature. Since the rotation of magnetization in the GaMnAs layer depends on magnetic free energy (which includes both Zeeman and anisotropy energies), magnetic anisotropy of the GaMnAs must be precisely determined at the value of the current involved in the experiment. Fortunately the techniques for determining magnetic anisotropy in ferromagnetic films are well developed through the angle-dependent planar Hall effect measurements and analyzing them in terms of the Stoner-Wohlfarth model,[2] as described in Refs. [3-5][3-5] For this purpose we performed systematic measurements of angular dependence of the planar Hall resistance (PHR) by using a series of different currents, and fitted the data using free energy minima conditions. The PHR data obtained with six different currents are plotted in Fig. S1, along with the fitting results.

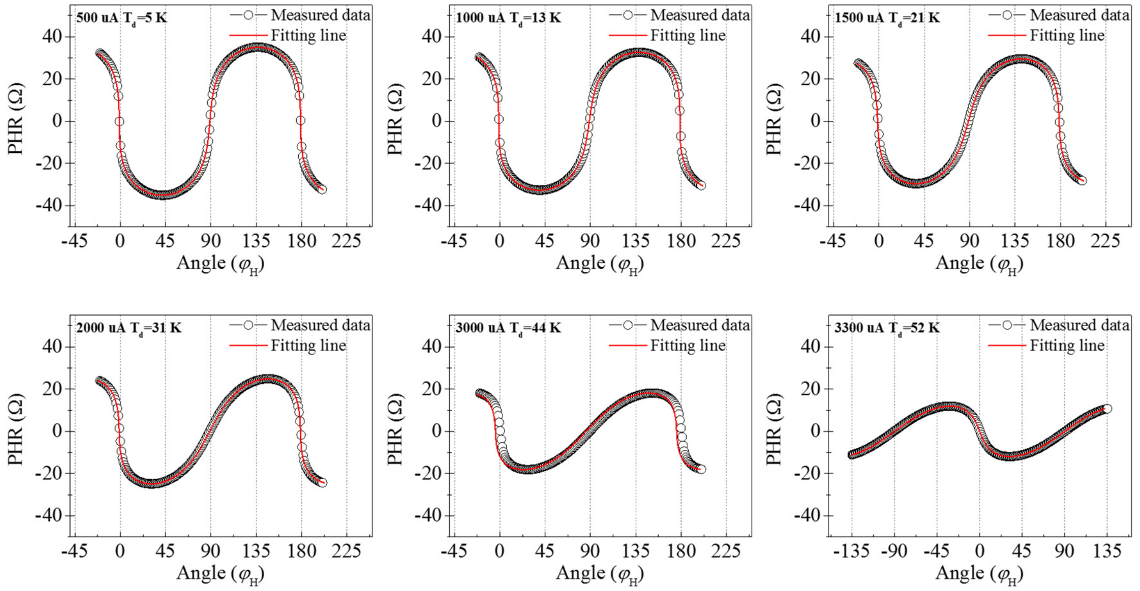

Fig. S1. Planar Hall resistance data obtained with six different currents by rotating an applied magnetic field in the (001) plane of the GaMnAs layer (open circles). Corresponding theoretical fits are shown as solid curves.

Magnetic anisotropy fields of the GaMnAs layer obtained by this fitting process for six current values are shown in Table S1. The results show that the cubic anisotropy field $\mathbf{H_c}$ rapidly decreases with increasing magnitude of the applied current, while the uniaxial anisotropy field $\mathbf{H_u}$ is less sensitive to the current. This anisotropy behaviour is closely similar to that observed with increasing temperature, indicating effects of Joule heating as discussed in Supplementary Material 3. Device temperatures T_d corresponding to the applied currents, as obtained in Supplementary Material 3, are shown in the second column of Table S1.

Table. S1. Magnitude of current, device temperature, and magnetic anisotropy parameters

Current (mA) (Current density)	Device A T_d (K)	Magnetic anisotropy field (Oe)	
		$\mathbf{H_C}$	$\mathbf{H_U}$
0.5 mA (0.2×10^5 A/cm^2)	5	1762 ± 8	91 ± 2
1.0 mA (0.4×10^5 A/cm^2)	13	1171 ± 4	110 ± 1
1.5 mA (0.6×10^5 A/cm^2)	21	656 ± 2	129 ± 0.5
2.0 mA (0.8×10^5 A/cm^2)	31	296 ± 2	134 ± 0.4
3.0 mA (1.2×10^5 A/cm^2)	44	136 ± 1	118 ± 0.3
3.3 mA (1.32×10^5 A/cm^2)	52	69 ± 1	93 ± 0.3

Free energy diagrams constructed by using these anisotropy fields are plotted in Fig. S2. While the four energy barriers are clear at the <110> directions for the energy diagram obtained with small currents, their heights are significantly reduced for larger currents, and the shape of the energy diagrams gradually becomes dumbbell-like as the current increases. This indicates significant Joule heating by the current in our Hall device.

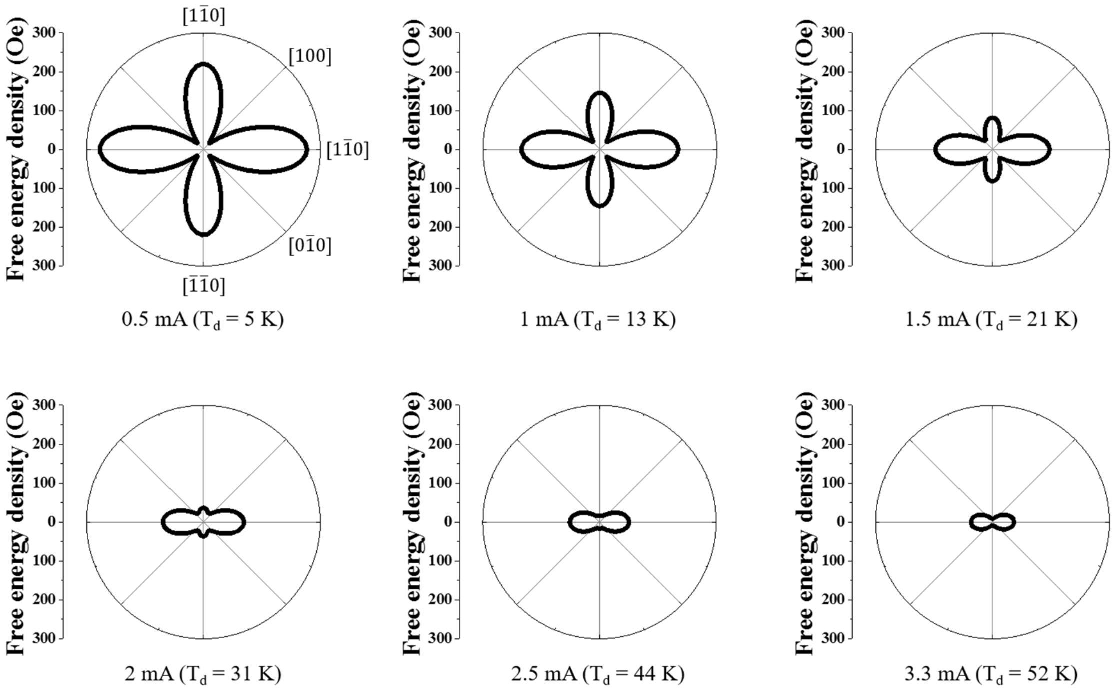

Fig. S2. Magnetic free energy diagrams obtained for six different current magnitudes used in measuring PHR shown in Fig. S1. The current used in the measurement and the corresponding effective temperature of the device are given below each diagram.

Supplementary 2

In order to achieve full rotation of magnetization in both GaMnAs and Fe layers, we used a larger magnetic field (sufficient to overcome the energy barriers in the Fe film) to achieve complete magnetization reversals in both layers. Magnetizations of both the A and B device were initialized using a strong magnetic field at the same orientation as that used for Fig. 2 in the main text. The field was then reduced to 100 Oe and rotated over 360 degrees, as PHR was measured as a function of angle. The results are shown in Fig. S3, where open symbols are used for PHR results obtained with CW rotation, and solid symbols for CCW rotation. The PHR behaviour for device A during field rotation is nearly the same as that

observed with the 23 Oe, as seen in Fig. 2 in the main text. However, the behaviour of magnetization for device B looks very different. Specifically, in addition to transitions of magnetization at φ_H= 0°, φ_H= 180° and φ_H= 360° originating from reorientations of magnetization in the GaMnAs layer, there are now distinct sharp transitions at positions slightly away from φ_H= 0°, φ_H= 90°, φ_H= 180°, φ_H= 270°, and φ_H= 360°. Importantly, these transitions occur at larger angles for the CCW field rotation, and at smaller angles for the CW rotation. These transitions, showing distinct hystereses, are caused by the Fe layer, which has two orthogonal in-plane magnetic easy axes due to its cubic anisotropy shown in blue in Fig. S3(c). Magnetization orientations occurring during the CCW rotation of the field are shown as solid red arrows in Fig. S3(b) for the GaMnAs layer, and as open blue arrows for the Fe layer. As one can see from the directions of the respective arrows, the magnetizations of both GaMnAs and Fe rotate over 360° when the field is rotated. This indicates that the magnetization in the Fe layer now makes transitions across the four energy barriers at each of the <110> directions as the field of 100 Oe is rotated over 360°.

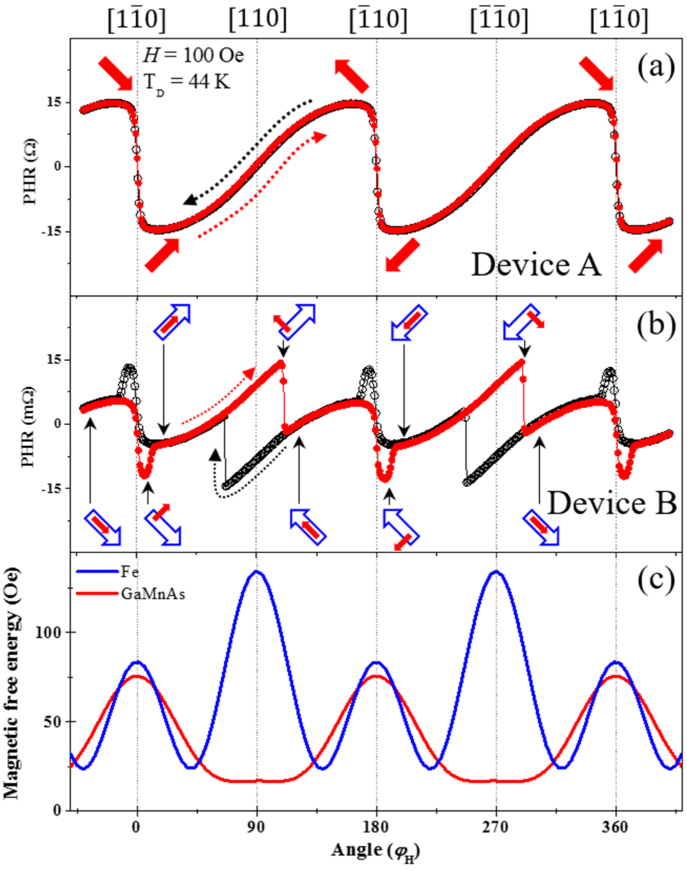

Fig. S3. PHR data measured with a field strength of 100 Oe. Data in the panels of the first and the second rows are obtained using device A and B, respectively. The red (solid) and black (open) symbols represent data taken

with CCW and CW rotation of the field, respectively. Directions of magnetization are shown with red (solid) for the GaMnAs layer and blue (open) arrows for the Fe layer at corresponding angular positions. Magnetic free energy density profiles obtained for GaMnAs (red curves) and Fe layers (blue) by using currents of 3.00 mA are shown in panels (c).

Supplementary 3

Since the magnetoresistance (MR) and the planar Hall resistance (PHR) of the GaMnAs layer show sensitive dependences on temperature, the effect of Joule heating by current can be investigated by measuring these properties. We first measured the temperature dependences of the MR and the PHR for device A with a current density of 0.2 mA, which is sufficiently small not to generate any detectable Joule heating during the measurement. These data are plotted with black curves in Fig. S4, where the upper and lower panels represent MR and PHR, respectively. We then repeated the same MR and PHR measurements by systematically changing the magnitude of the current while the temperature of the sample holder was kept at 3 K during the measurement. MR and PHR measured with different currents are plotted with red solid circles in upper and lower panels of Fig. S4. During these measurements, a background magnetic field of 1000 Oe was applied along the $[0\bar{1}0]$ direction of the GaMnAs layer to eliminate any changes in MR or in PHR that might arise from relaxation of magnetization due to changes in magnetic anisotropy as the temperature changes.

The plots show that both the MR and the PHR increase monotonically as the current increases from 0.4 mA to 3.5 mA. The values of the MR and the PHR measured at each current can be mapped into those obtained by varying the temperature. Such mapping provides the effective temperature T_d of our Hall device at different values of applied current, as plotted in the inset of Fig. S4. It is clear that T_d of the device monotonically increases over 50 K as the current increases from 0.4 mA to 3.5 mA. This implies that the process of magnetization reversal in the device will be different at different values of the current due to differences in magnetic anisotropy of the GaMnAs layer induced by Joule heating. For this reason we investigated magnetic anisotropy of the GaMnAs layer (Device A) by using several different current magnitudes, as described in the Supplementary Material 1.

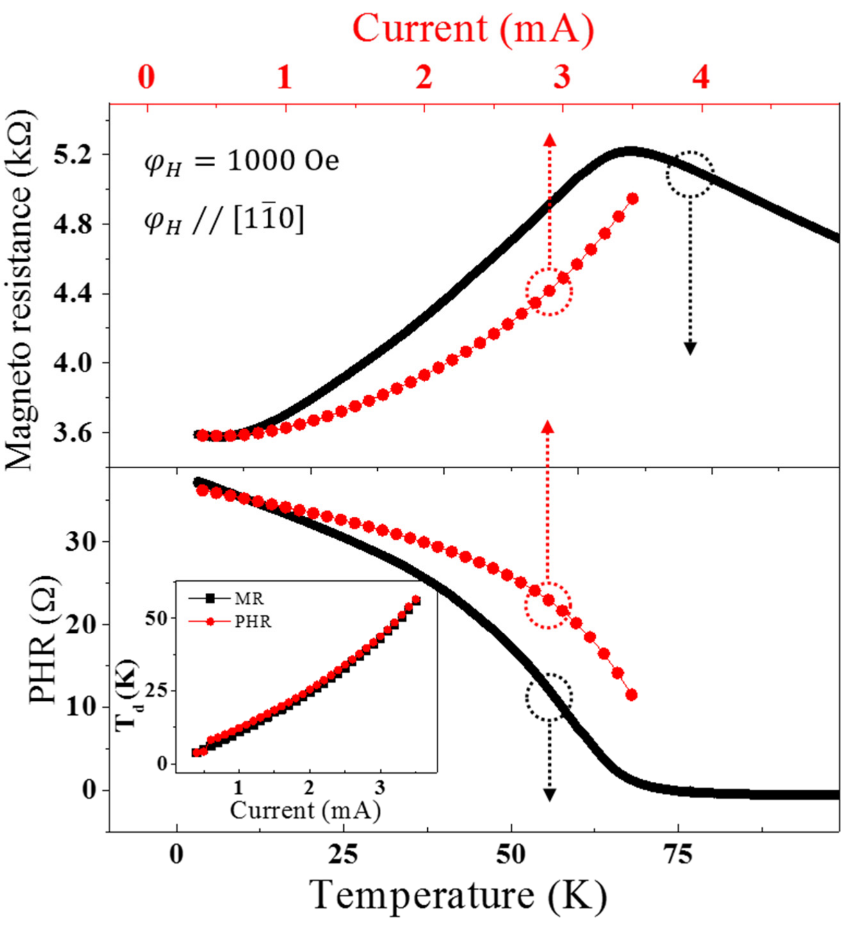

Fig. S4. Dependences of the MR (upper panel) and the PHR (lower panel) on magnitude of current (upper scale) and on temperature (lower scale). Inset of lower panel shows the dependence of device temperature, T_d, on the current.

Supplementary 4

In order to see the current dependence of the SOI field, we performed magnetization reversal experiment by using several difference currents, from which the strength of the SOI field was then determined. Figure S5 shows the data summary for the SOI field as a function of current density. The data in insets show PHR hystereses formed by currents of opposite polarity at two representative current densities. It is clear that the width of the hysteresis is larger for the larger current, indicating an increase of the SOI field with increasing current. The data in Fig. S5 also shows a linear dependence of the SOI field on the current density.

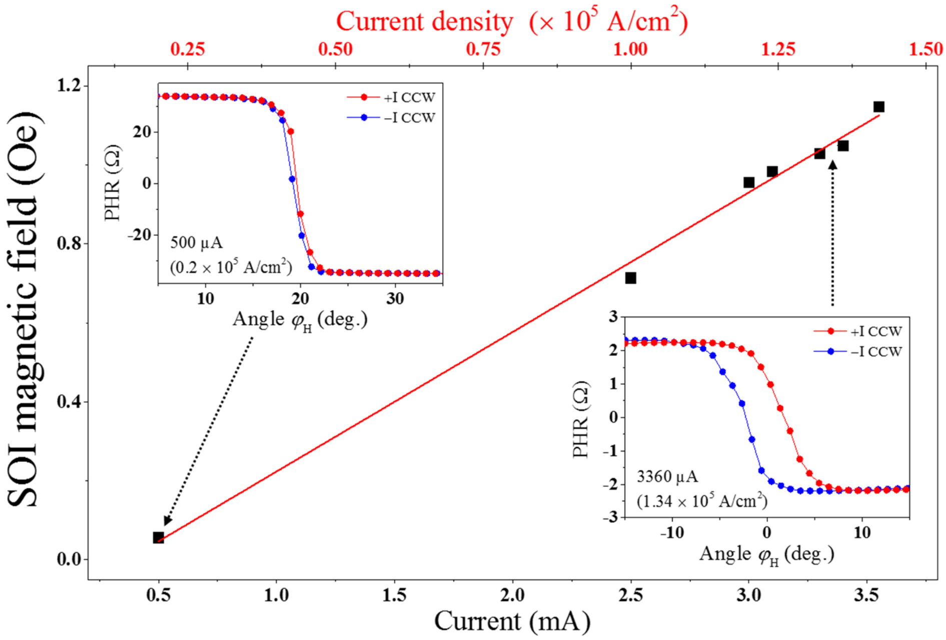

Fig. S5. Dependence of SOI field on current density obtained in our device. Insets show PHR hysteresis formed by opposite polarities of currents at two representative magnitudes.

An alternative method for obtaining the SO-field in our GaMnAs film is by carrying out 2nd harmonic Hall measurements,[6] which directly provide the strength of the field. The 1st and the 2nd harmonic signals measured with several different currents during the field scan along the out-of-plane direction are plotted in the insets of Fig. S6. By using the equation $H_{\mathrm{SOI}} = -2\frac{C_{2\omega}}{dC_{\omega}/dH}$ developed in Ref. [6], we are able to obtain the SO-field plotted as black squares in Fig. S6. As seen in the figure, the SO-field obtained via the 2nd harmonic measurements also increases with increasing current density. The range of the SO-field obtained with 2nd harmonic measurement for the current density of ~ 10^5 A/cm^2 is consistent with the results obtained by the measurements of the angular dependence of PHR shown in Fig. S5.

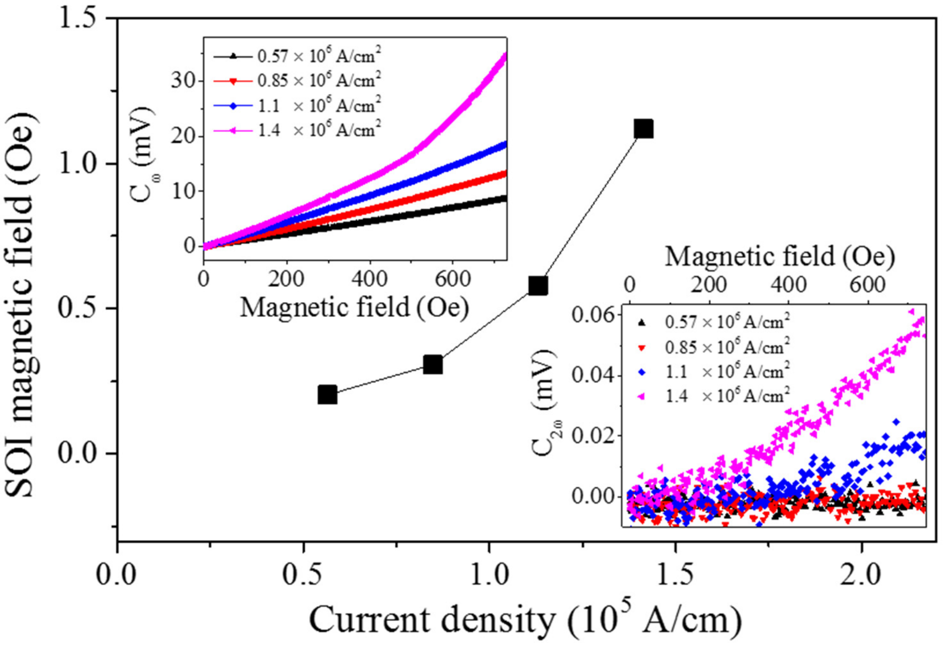

Fig. S6. Dependence of SOI field on current density obtained by 2nd harmonic measurements. Insets show 1st and the 2nd harmonic signals measured with few different currents.

Supplementary 5

We performed magnetization switching experiments as a function of applied current in a constant background field of H = 20 Oe applied along the $[1\bar{1}0]$ direction. Figure S7 shows the PHR data measured while the applied current was scanned between -3.0 mA and +3.0 mA. The data show a clear hysteresis, indicating a reorientation of magnetization as the current is swept. The PHR is seen to switch sign near $\pm$2.9 mA, which corresponds to a critical current that produces the reorientation of magnetization in the GaMnAs layer. As seen from the presence of intermediate values of PHR within the transition region, in that region the GaMnAs layer is characterized by a multi-domain landscape.

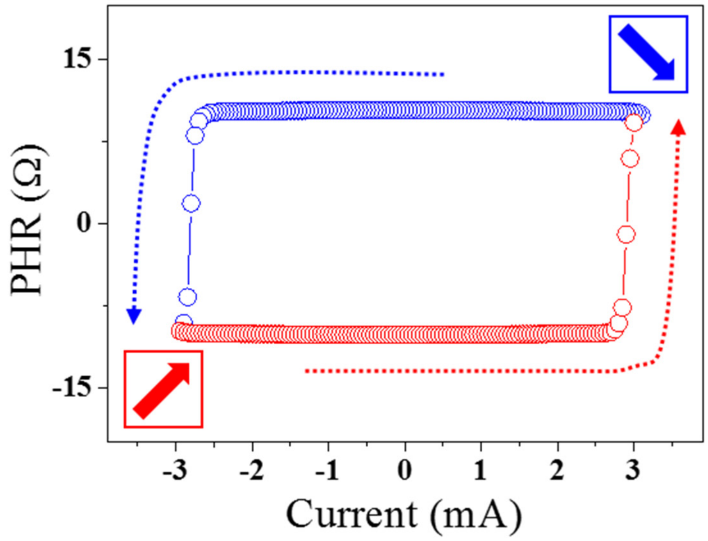

Fig. S7. PHR data obtained while the applied current is swept between -3.0 mA and +3.0 mA. Up and down current scans are plotted with blue and red symbols, respectively. Reorientation of magnetization occurs near the critical current of |2.9 mA|.

Supplementary 6

The presence of interlayer exchange coupling can be verified by minor loop scan experiments, in which the magnetization of only one layer switches, while that of the other layer remains unchanged. Figure S8 shows PHR data obtained from major (black squares) and minor loop scans (open and solid red circles for CW and CCW rotations of the applied field, respectively). As seen from the directions of magnetization shown by arrows, the hysteresis in the minor loop scan arises from the reorientation of magnetization only in the GaMnAs layer, while that of Fe layer is fixed. If an exchange coupling between Fe and GaMnAs layers were present, the hysteresis would shift.[7,8] However, the observed hysteresis does not show shift, as seen in Fig. S8, thus indicating the absence of noticeable interlayer exchange coupling between the Fe and GaMnAs layers.

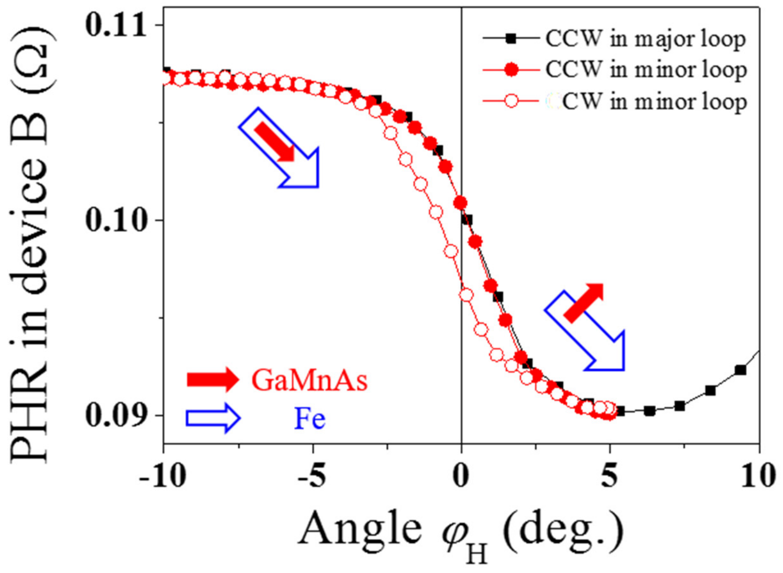

Fig. S8. PHR data obtained from major (black squares) and minor loop scan, in which reorientation of magnetization occurs only in the GaMnAs layer while that of Fe layer is fixed as shown by tick arrows. The data plotted with open and solid red circles are obtained with CW and CCW rotation, respectively. The hysteresis of minor loop does not show shift indicating the absence of the interlayer exchange coupling between Fe and GaMnAs layers.

References

1 Shin, D. Y., Chung, S. J., Lee, S., Liu, X. & Furdyna, J. K. Temperature dependence of magnetic anisotropy in ferromagnetic (Ga,Mn)As films: Investigation by the planar Hall effect. *Phys. Rev. B* **76**, 035327 (2007).

2 Stoner, E. C. & Wohlfarth, E. P. A mechanism of magnetic hysteresis in heterogeneous alloys. *Philos. Trans. R. Soc. London, Ser. A* **240**, 599-642 (1948).

3 Okamoto, K. A New Method for Analysis of Magnetic-Anisotropy in Films Using the Spontaneous Hall-Effect. *J. Magn. Magn. Mater.* **35**, 353-355, doi:Doi 10.1016/0304-8853(83)90539-5 (1983).

4 Liu, X. *et al.* Perpendicular magnetization reversal, magnetic anisotropy, multistep spin switching, and domain nucleation and expansion in Ga1-xMnxAs films. *J. Appl. Phys.* **98**, 063904 (2005).

5 Son, H. *et al.* Quantitative investigation of the magnetic anisotropy in GaMnAs film by using Hall measurement. *J. Appl. Phys.* **103**, 07F313 (2008).

6 Pi, U. H. *et al.* Tilting of the spin orientation induced by Rashba effect in ferromagnetic metal layer. *Appl. Phys. Lett.* **97**, doi:Artn 162507
10.1063/1.3502596 (2010).

7 Ge, Z. *et al.* Magnetization reversal in (Ga,Mn)As/MnO exchange-biased structures: Investigation by planar Hall effect. *Phys. Rev. B* **75**, 014407, doi:Artn 014407
Doi 10.1103/Physrevb.75.014407 (2007).

8 Alsmadi, A. M. *et al.* Interfacial exchange coupling in Fe/(Ga,Mn)As bilayers. *Phys. Rev. B* **89**, 224409, doi:Artn 224409
10.1103/Physrevb.89.224409 (2014).
